# Analysis of Cytokine Levers in Pleural Effusions of Tuberculous Pleurisy and Tuberculous Empyema

**DOI:** 10.1155/2016/3068103

**Published:** 2016-02-29

**Authors:** Lei Yang, Yan-Jie Hu, Fu-Gen Li, Xiu-Jun Chang, Tian-Hui Zhang, Zi-Tong Wang

**Affiliations:** ^1^Department of Thoracic Surgery, Beijing Chest Hospital, Capital Medical University, Beijing 101149, China; ^2^Tuberculosis Immunology Department, Beijing Chest Hospital, Capital Medical University, Beijing 101149, China

## Abstract

The aim is to examine whether the interleukin-1*β* (IL-1*β*), IL-2, IL-6, tumor necrosis factor-*α* (TNF-*α*), plasminogen activator inhibitor type-1 (PAI-1), and tissue plasminogen activator (t-PA) levels were different in pleural effusions of tuberculous pleurisy and tuberculous empyema. IL-1*β*, IL-2, IL-6, TNF-*α*, PAI-1, and t-PA levels in pleural fluids of 40 patients with tuberculous pleurisy and 38 patients with tuberculous empyema were measured. The levels of IL-1*β*, PAI-1, and t-PA in the pleural effusions were different between tuberculous pleurisy and tuberculous empyema; it could be helpful to differentiate the two diseases. The levels of PAI-1, IL-1*β* were higher and t-PA, IL-6 were lower in pleural effusions of the patients with tuberculous empyema and who must undergo operation than the patients who could be treated with closed drainage and anti-TB chemotheraphy. These indications may be helpful to evaluate whether the patient needs the operation.

## 1. Introduction

Pleura is divided into a parietal layer which lines the inner aspect of the chest wall and a visceral layer which covers the interlobar fissures. The mean amount of pleural fluid in the normal is as small as 8.4 ± 4.3 mL. Fluid that enters the pleural space can originate in the pleural capillaries, the interstitial spaces of the lung, the intrathoracic lymphatics, the intrathoracic blood vessels, or the peritoneal cavity. Pleural fluid is usually absorbed through the lymphatic vessels in the parietal pleura by means of stomas in the parietal pleura, or through the alternative transcytosis [1].

Despite being isolated by Robert Koch in 1882, as well as the availability of effective treatment and the use of a live attenuated vaccine in many parts of the world, TB remains a serious health issue. In 2013, an estimated 9 million people developed active TB, with 1.5 million deaths attributed to the disease. According to the World Health Organization the incidence of pulmonary TB in some regions is as high as 1,000 cases per 100,000 persons [2]. Although TB affects the lungs in the majority of patients, extrapulmonary TB serves as the initial presentation in about 25% of adults and primarily involves the lymph nodes and pleura [3]. Tuberculosis-related pleural effusions occur in approximately 2% to 10% of TB patients, with a male to female ratio of 2 : 1 and can result from primary or reactive TB [3, 4]. Even with regular treatment, tuberculous pleurisy may develop to tuberculous empyema, a kind of chronic and fatal sequelae.

Previous studies have indicated that levels of some cytokines in pleural effusions were related to the disease. We aimed to examine whether the interleukin-1*β* (IL-1*β*), IL-2, IL-6, tumor necrosis factor-*α* (TNF-*α*), plasminogen activator inhibitor type-1 (PAI-1), and tissue plasminogen activator (t-PA) levels were different in pleural effusions of tuberculous pleurisy and tuberculous empyema.

## 2. Materials and Methods

The study was approved by our institutional review board. The study group consisted 40 patients with tuberculous pleurisy and 38 patients with tuberculous empyema. All the patients were diagnosed and treated in Beijing Chest Hospital, Capital Medical University. Thoracal ultrasonograms were obtained from patients thought to have pleural effusions in consideration of medical history, physical examination, and chest X-rays. The presence and the amount of pleural fluids were determined and then thoracocentesis was performed.

The diagnosis of tuberculous pleurisy was made if there was at least one of the following criteria: (1) the detection of* Mycobacterium tuberculosis* in pleural fluid by acid-fast bacilli (AFB) smear and/or culture; (2) typical histologic findings of a pleural biopsy specimen; (3) the presence of active pulmonary tuberculosis; and (4) typical clinical and pleural fluid findings that could exclude other causes (lymphocyte dominant and an ADA level >60 IU/L) in cases without bacteriologic or histologic confirmation [4].

Empyema was established by one or more of the following criteria: purulent fluid on macroscopic examination, positive Gram stain, and/or microorganism growth in culture and pH < 7.2 or glucose < 40 mg/dL [5]. A diagnosis of tuberculous empyema was made if there was at least one of the following criteria: (1) pleural fluid smear and/or culture was positive for acid-fact bacilli (AFB)/*Mycobacterium tuberculosis* on two or more occasions; (2) radiological evidence of active pulmonary tuberculosis on CT scans (nodular consolidation in apical segments/tree-in-bud appearance/mediastinal lymphadenopathy with central necrosis); and (3) concomitant positive sputum smears for AFB [6].

The IL-1*β*, IL-2, IL-6, TNF-*α*, PAI-1, and t-PA levels were measured using enzyme-linked immunosorbent assay (ELISA) commercial kits (R&D, American). Statistical evaluations between different groups were done with Wilcoxon rank sum test using SPSS 16.0 software programs. A *P* value less than 0.05 was considered to be statistically significant.

## 3. Results

There were 40 patients with tuberculous pleurisy and 38 patients with tuberculous empyema in the current series. The age at the time of diagnosis ranged from 22 to 59 years (mean age was 43.1 years). Among the patients with tuberculous empyema, 23 patients were treated with closed drainage and anti-TB chemotheraphy; the other 15 patients needed operation because the lung could not reexpand after closed drainage.

The IL-1*β*, IL-2, IL-6, TNF-*α*, PAI-1, and t-PA levels in the patients with tuberculous pleurisy and tuberculous empyema were displayed in Table 1. The levels of IL-1*β*, PAI-1, and t-PA were different in the two groups.

The IL-1*β*, IL-2, IL-6, TNF-*α*, PAI-1, and t-PA levels in the patients with tuberculous empyema treated by closed drainage and operation were displayed in Table 2. The levels of IL-1*β*, IL-6, PAI-1, and t-PA were different in the two groups.

## 4. Discussion

Tuberculous pleurisy is the first or second most common form of extrapulmonary tuberculosis as well as the main cause of pleural effusion in many countries [7]. Rupture of a subpleural caseous focus in the lung into the pleural space is thought to be the initial event in the pathogenesis of primary tuberculous pleurisy. Mycobacterial antigens enter the pleural space and interact with T-cells previously sensitized to mycobacteria and then result in a delayed hypersensitivity reaction [8, 9]. Tuberculous empyema is less common and represents a distinct entity of chronic, active infection within the pleural space. It is characterised by purulent fluid where virtually all the nucleated white blood cells are neutrophils and can occur in several settings: (I) progression of a primary TB pleuritis; (II) direct extension of infection into the pleural space from thoracic lymph nodes or a subdiaphragmatic focus; (III) haematogenous spread; or (IV) following pneumonectomy [10]. Because of the inflammatory response and immune reaction, some cytokines may be secreted into the pleural effusions and may be different between the two diseases. Some patients with early stage tuberculous empyema could be treated with closed drainage and anti-TB chemotheraphy, but some patients must undergo operation because the lung could not reexpand. We also wanted to know whether the levels of the cytokines in the two groups' patients were different. As far as we knew, there are few reports of this issue.

IL-1 consists of 2 homologous cytokines, IL-1*α* and IL-1*β*, predominantly produced by macrophages [11]. Both *α* and *β* subtypes of IL-1 can bind to 2 receptors. IL-1RI is the biological active receptor involved in signal transduction, whereas IL-1RII operates as a nonsignaling decoy receptor [12]. IL-1 plays a major role in driving and sustaining chronic inflammation associated with a series of autoimmune and metabolic disorders [13, 14]. More recently, it has become clear that IL-1*β* is also of critical importance for host control of TB infection given that mice deficient in IL-1R or its adaptor MyD88 succumb rapidly to low-dose aerosol infection with TB [15, 16]. Previous research indicated that the levels of IL-1*β* were lower in the pleural effusions of tuberculous pleurisy than empyema [17]. Our results were similar to it. Moreover, our results also indicated that the IL-1*β* levels of pleural effusions of the patients with tuberculous empyema and who must undergo operation were higher than the patients who could be treated with closed drainage and anti-TB chemotheraphy.

IL-2 levels in pleural effusions due to various diseases had been reported. The previous reports had stated that the IL-2 levels in pleural effusions of cancer and empyema patients were lower than tuberculous pleurisy patients [17–19]. In our study the IL-2 levels in the pleural effusion of patients with tuberculous pleurisy and tuberculous empyema had no statistical difference, as well as the two groups of tuberculous empyema.

Various researches had been performed concerning the levels of IL-6 which is the mediator and the regulator of inflammatory responses in pleural effusions due to miscellaneous diseases. Xirouchaki et al. reported the levels of IL-6 in pleural fluids to be significantly higher in the exudate group rather than the transudate group and the IL-6 concentrations significantly higher in the tuberculous group rather than the parapneumonic effusion group [20, 21]. Akarsu et al. found statistically significant differences between the empyematous and the tuberculous pleural effusion groups [17]. In our study the IL-6 levels in the pleural effusion of patients with tuberculous pleurisy and tuberculous empyema had no statistical difference, but the levels of pleural effusions of the patients with tuberculous empyema and who must undergo operation were lower than the patients who could be treated with closed drainage and anti-TB chemotheraphy.

TNF-*α* is an important cytokine involved in acute pleural inflammation leading to the development of parapneumonic effusion [22, 23]. Previous reports showed the TNF-*α* levels in empyema and complicated parapneumonic effusions compared to malignant effusions, whereas tuberculous pleural fluid showed the highest TNF-*α* level [24, 25]. In our study the TNF-*α* levels in the pleural effusion of patients with tuberculous pleurisy and tuberculous empyema had no statistical difference, as well as the two groups of tuberculous empyema.

There are few reports about the IL-2, IL-6, and TNF-*α* levels in pleural effusions of patients with tuberculous pleurisy and tuberculous empyema.

Previous studies show the levels of the three cytokines in pleural effusions were different between empyema and tuberculous pleurisy, but our results showed had no different between tuberculous pleurisy and tuberculous empyema. We thought it may be attributed to the difference between tuberculous empyema and the empyema due to other bacteria.

Plasminogen activator inhibitor type-1 (PAI-1) and tissue plasminogen activator (t-PA) are important cytokines of fibrinolytic system and play a vital role in activation and inhibition, respectively. An imbalance in fibrinolytic system activation/inhibition in the pleural space could account for the abnormal fibrin turnover seen in some pleural effusions [26, 27]. Some authors reported that PAI levels were increased and t-PA levels decreased in empyema. They thought it could partially contribute to the higher pleural fibrin deposition seen in the effusion and could be related to the development of clinical complications of empyema [25]. Our results were similar to the previous reports. The levels of PAI-1 were lower and t-PA were higher in the pleural effusions of tuberculous pleurisy than empyema. Moreover, our results also indicated that the PAI-1 were higher and t-PA were lower in pleural effusions of the patients with tuberculous empyema and who must undergo operation than the patients who could be treated with closed drainage and anti-TB chemotheraphy.

This study has showed that the levels of IL-1*β*, PAI-1, and t-PA in the pleural effusions were different between tuberculous pleurisy and tuberculous empyema; it could be helpful to differentiate the two diseases. The levels of PAI-1, IL-1*β* were higher and t-PA, IL-6 were lower in pleural effusions of the patients with tuberculous empyema and who must undergo operation than the patients who could be treated with closed drainage and anti-TB chemotheraphy. These indications may be helpful to evaluate whether the patient needs the operation.

## Figures and Tables

**Table 1:** IL-1*β*, IL-2, IL-6, TNF-*α*, PAI-1, and t-PA levels in the patients with tuberculous pleurisy and tuberculous empyema.

	IL-1*β* (ng/mL)	IL-2 (pg/mL)	IL-6 (ng/mL)	TNF-*α* (pg/mL)	PAI-1 (ng/mL)	t-PA (ng/mL)
Tuberculous pleurisy (*n* = 40)	0.3073	320.32	108.32	105.18	1.5035	0.2121
Tuberculous empyema (*n* = 38)	0.6709	313.12	110.80	91.58	1.8378	0.1461
*Z*	−3.539	−0.500	−0.010	−0.110	−2.784	−3.470
*P*	0.0004	0.6172	0.9922	0.9124	0.0054	0.0005

**Table 2:** IL-1*β*, IL-2, IL-6, TNF-*α*, PAI-1, and t-PA levels in the patients with tuberculous empyema treated by closed drainage and operation.

	IL-1*β* (ng/mL)	IL-2 (pg/mL)	IL-6 (ng/mL)	TNF-*α* (pg/mL)	PAI-1 (ng/mL)	t-PA (ng/mL)
Closed drainage (*n* = 23)	0.4347	336.93	110.55	108.83	1.6467	0.1726
Operation (*n* = 15)	1.0757	276.55	91.11	65.14	2.1308	0.1126
*Z*	−1.9263	−1.3588	−1.8044	−1.2394	−2.8376	−02.365
*P*	0.0270	0.1742	0.0356	0.2152	0.0045	0.017
